# Body mass index and baseline platelet count as predictive factors in Merkel cell carcinoma patients treated with avelumab

**DOI:** 10.3389/fonc.2023.1141500

**Published:** 2023-04-17

**Authors:** Lorena Incorvaia, Alessandra Dimino, Laura Algeri, Chiara Brando, Luigi Magrin, Ida De Luca, Erika Pedone, Alessandro Perez, Roberta Sciacchitano, Annalisa Bonasera, Tancredi Didier Bazan Russo, Federica Li Pomi, Marta Peri, Valerio Gristina, Antonio Galvano, Dario Giuffrida, Ivan Fazio, Francesca Toia, Adriana Cordova, Ada Maria Florena, Antonio Giordano, Viviana Bazan, Antonio Russo, Giuseppe Badalamenti

**Affiliations:** ^1^ Department of Surgical, Oncological and Oral Sciences, Section of Medical Oncology, University of Palermo, Palermo, Italy; ^2^ Department of Oncology, Istituto Oncologico del Mediterraneo, Catania, Italy; ^3^ Department of Clinical and Experimental Medicine, Section of Dermatology, University of Messina, Messina, Italy; ^4^ Radiotherapy Unit, Clinica Macchiarella, Palermo, Italy; ^5^ Division of Plastic and Reconstructive Surgery, Department of Surgical, Oncological and Oral Sciences, University of Palermo, Palermo, Italy; ^6^ Pathologic Anatomy Unit, Department of Health Promotion, Mother and Child Care, Internal Medicine and Medical Specialties, University of Palermo, Palermo, Italy; ^7^ Sbarro Institute for Cancer Research and Molecular Medicine, Center for Biotechnology, College of Science and Technology, Temple University, Philadelphia, PA, United States; ^8^ Department of Biomedicine, Neuroscience and Advanced Diagnostics (Bind.), Section of Medical Oncology, University of Palermo, Palermo, Italy; ^9^ Institute for Cancer Research and Molecular Medicine and Center of Biotechnology, Temple University, Philadelphia, PA, United States

**Keywords:** Merkel cell carcinoma (MCC), avelumab, body mass index - BMI, predictive factors, immunotherapy, skin cancer non melanoma

## Abstract

**Background:**

Merkel cell carcinoma (MCC) is a rare and aggressive skin cancer, associated with a worse prognosis. The Immune Checkpoint Inhibitors (ICIs) avelumab and pembrolizumab have been recently approved as first-line treatment in metastatic MCC (mMCC). The clinical observation of improved outcomes in obese patients following treatment with ICIs, known as the “obesity paradox”, has been studied across many types of tumors. Probably due to the rarity of this tumor, data on mMMC patients are lacking.

**Patients and methods:**

This is an observational, hospital-based, study to investigate the role of Body Mass Index (BMI) as predictive biomarker of ICI response in mMCC patients treated with avelumab as first-line treatment. The study population included the patients treated from February 2019 to October 2022 in an Italian referral center for rare tumors. Clinico-pathological characteristics, BMI, laboratory parameters (NLR and platelet count), and response to avelumab were analyzed from a MCC System database prospectively collected.

**Results:**

Thirty-two (32) patients were included. Notably, the presence of pre-treatment BMI ≥ 30 was significantly associated with longer PFS [BMI < 30 Group: median PFS, 4 months (95% CI: 2.5-5.4); BMI ≥ 30 Group: median PFS, not reached; p<0.001)[. Additionally, the median PFS was significantly higher in patients with higher PLT (median PFS: 10 months in the “low PLT” Group (95% CI: 4.9, 16.1) vs 33 months (95% CI: 24.3, 43.2) in the “high PLT” Group (p=0.006). The multivariable Cox regression model confirmed these results.

**Conclusion:**

To our knowledge, this is the first study that investigates the predictive role of BMI in MCC patients. Our data were consistent with the clinical observation of improved outcomes in obese patients across other tumor types. Thus, advanced age, a weakened immune system, and the obesity-associated “inflammaging”, are key factors that could impact the cancer immune responses of mMCC patients.

## 1 Introduction

Merkel Cell Carcinoma (MCC) is a rare primary cutaneous neuroendocrine carcinoma usually involving the sun-exposed skin of elderly individuals, but sometimes also the trunk and limbs (1). Immunosuppression, chronic ultraviolet (UV) exposure, and old age are known predisposing factors (2), and MMC incidence is rising in Western countries (3). Clinical presentation is a rapidly growing, painless, erythematous/violaceous nodule or plaque (4). MCC is an aggressive tumor, as synchronous nodal and/or systemic metastases are present in about one-third of cases (2). Merkel Cell Polyomavirus (MCPyV) seems to be the pathogenic factor for about 80% of MMCs in the northern hemisphere, as viral DNA was shown to be integrated within the tumor genome (1, 5). The genetic damage induced by UV radiation accounts for the pathogenesis of the MCPyV-negative MCCS, showing a high tumor mutational burden (TMB) and a UV mutational signature (6).

The Immune Checkpoint Inhibitors (ICIs), anti-PD-L1 avelumab and anti-PD-1 pembrolizumab, have been recently approved as first-line treatment in metastatic MCC, although only a minority of patients exhibit long-term response (7, 8). Response to ICI therapy did not correlate either with MCPyV or with UV mutational signature status (9). An association with PD-1/PD-L1 tumor tissue expression was found (9, 10), but current assays remain imperfect to predict which patient will benefit from immunotherapy (11). Thus, further predictors of response are needed.

Recently, the clinical, contrasting, observation of improved outcomes in obese patients following treatment with immune checkpoint blockade, known as the “obesity paradox”, has been studied across many types of tumors (12). Cancer patients with high body mass index (BMI) seem to respond better to ICI therapy when compared to those with normal BMI (13–17). Probably due to the rarity of this tumor, data on the relationship between BMI and response to ICI treatment among MMC patients are lacking. Although the mechanistic link between metabolic state and immunotherapy benefit was not clearly elucidated, clinical data linking excess adiposity to ICI response in MCC patients could give more insights into the complex immune-metabolic interactions, serving as both predictive and stratification factors in future clinical trials.

## 2 Methods

### 2.1 Study design and patient selection

This was an observational, hospital-based, study to investigate the role of BMI as a predictive biomarker of immunotherapy response in metastatic MCC (mMCC) patients treated with the ICI avelumab.

The study population included adult patients with a pathologically confirmed diagnosis of MCC and advanced disease, treated with avelumab in the first-line setting, according to medical choice and current therapeutic options. The patients were treated from February 2019 to October 2022 in an Italian referral center for rare tumors: the “Sicilian Regional Center for the Prevention, Diagnosis and Treatment of Rare and Heredo-Familial Tumors” of the University Hospital Policlinico “Paolo Giaccone” of Palermo. Patients data were analyzed from a MCC System database prospectively collected. The pathological information collected on primary MCC included the histology, the Ki-67 (%) value and chromogranin A expression from pathology reports for clinical use. The clinical data on disease stages, comorbidities and hepatopathy, second tumor history, the primary site of the tumors, number of metastatic sites, dose, and duration of avelumab treatment, were abstracted from the clinical records. Pre-treatment BMI, Neutrophil-to-Lymphocyte Ratio (NLR) and platelet count (PLT) were recorded. The NLR was extracted from the routinely performed blood cell count, as the absolute count of neutrophils divided by the absolute count of lymphocytes from peripheral blood samples collected at baseline. BMI was calculated as weight in kilograms divided by height in meters squared. Normal weight (BMI=18.5-24.9), overweight (BMI=25-29.9), and obesity (BMI ≥ 30) were classified based on the World Health Organization (WHO) recommendations (nota).

The tumor response [progressive disease (PD), stable disease (SD), partial response (PR), complete response (CR)] according to Response Evaluation Criteria In Solid Tumors (RECIST version 1.1.), objective response rate (ORR), progression-free survival (PFS) to avelumab treatment, and overall survival (OS) were assessed. The association between clinic-pathological variables, BMI, and clinical outcomes was evaluated. All clinical characteristics were anonymously recorded and coded.

The protocol was approved by the ethical committee of the University-affiliated Hospital A.O.U.P. “Paolo Giaccone” (approval number 1122), and the study was conducted in accordance with the Declaration of Helsinki and Good Clinical Practice guidelines.

### 2.2 Statistical considerations

Descriptive analyses were used to assess patients’ characteristics. The differences between subgroups were evaluated by Fisher’s exact test. Pearson’s correlation coefficient was used to define the relationship between age and BMI.

The primary outcome of the study was progression-free survival (PFS). PFS was calculated from the beginning of the immunotherapy treatment to death by any cause or disease progression or last follow-up (censored patients). Overall survival (OS) was calculated from the start of immunotherapy treatment to death by any cause or last follow-up (censored patients). The analysis of PFS and OS between groups was compared using the Kaplan-Meier method and log-rank test. The receiver operating characteristic (ROC) curves analysis was used to determine the optimal cut-off for PLT count and NLR.

To identify independent prognostic factors for PFS and OS, univariable and multivariable Cox proportional hazard regression models were built. P values <0.05 were considered statistically significant. Statistical analyses were conducted using IBM SPSS Statistics Version 29.0 (IBM Corporation, Armonk, NY, USA).

## 3 Results

### 3.1 Study population

From February 2019 to October 2022, thirty-two (32) patients were included in the study. The median age was 75 (range 43-89). Seventeen patients were men (53%) and fifteen were women (47%). All the patients had an ECOG Performance status of 0 or 1. Twenty-one patients (66%) had two or more comorbidities (cardiovascular, endocrinological and/or another concomitant disease). In particular, twelve (12) patients (38%) were obese (BMI ≥ 30), while twenty (20) patients had a BMI <30 (62%). Additionally, seven (7) patients had a previous hepatopathy and eight (8) patients had a second hematological or solid tumor. The most common tumor primary site was the lower limb (21 patients, 66%), followed by unknown origin (5 patients, 16%). Fifteen (15) patients had only lymph node involvement (47%), and 17 patients (53%) showed other metastatic sites (visceral or soft tissues, alone or along with lymph node metastasis). Pathological and clinical patient characteristics are summarized in **Table 1**.

**Table 1:** Patients’ and disease characteristics.

Characteristic	All patients No. (%)	BMI<30 No. (%)	BMI≥ 30 No. (%)	p-value
**No. of Patients (%)**	32 (100)	20 (62.5)	12 (37.5)	–
**Gender** Male Female	17 (53.1) 15 (46.1)	10 (50.0) 10 (50.0)	7 (58.3) 5 (41.7)	0.2
**Age (years),** **median (range)** <75 y ≥75 y	75 (43–89) 16 (50.0) 16 (50.0)	7 (35.0) 13 (65.0)	9 (75.0) 3 (25.0)	0.02
**ECOG** 0 1	23 (71.9) 9 (28.1)	15 (75.0) 5 (25.0)	8 (66.7) 4 (33.3)	0.2
**No of Comorbidities** <2 ≥2	11 (34.4) 21 (65.6)	6 (30.0) 14 (70.0)	5 (41.7) 7 (58.3)	0.4
**Hepatopathy** No Yes Unknown	22 (68.8) 7 (21.9) 3 (9.3)	13 (65.0) 5 (25.0) 2 (10.0)	9 (75.0) 2 (16.7) 1 (8.3)	0.3
**Second Tumor** No Yes	24 (75.0) 8 (25.0)	14 (70.0) 6 (30.0)	10 (83.3) 2 (16.7)	0.7
**Primary site** Head and neck Trunk Upper limb Lower limb Unknown	2 (6.3) 2 (6.3) 2 (6.3) 21 (65.6) 5 (15.6)	1 (5.0) 0 (0.0) 2 (10.0) 14 (70.0) 3 (15.0)	1 (8.3) 2 (16.7) 0 (0.0) 7 (58.3) 2 (16.7)	0.03
**KI67%** <90 ≥90 Unknown	11 (34.4) 17 (53.1) 4 (12.5)	6 (30.0) 11 (55.0) 3 (15.0)	5 (41.7) 6 (50.0) 1 (8.3)	0.4
**Chromogranin-A** Negative Positive Not specified	1 (3.1) 23 (71.9) 8 (25.0)	0 (0.0) 14 (70.0) 6 (30.0.0)	1 (8.3) 9 (75.0) 2 (16.7)	0.7
**Metastatic at diagnosis** No Yes	13 (40.6) 19 (59.4)	10 (50.0) 10 (50.0)	3 (25.0) 9 (75.0)	0.1
**N. of metastatic sites** <2 ≥2	12 (37.5) 20 (62.5)	8 (40.0) 12 (60.0)	4 (33.3) 8 (66.7)	0.7
**Common site of metastasis** Lymph nodes Others	15 (46.9) 17 (53.1)	10 (50.0) 10 (50.0)	5 (41.7) 7 (58.3)	0.6
**NLR** <2, ≥2 Unknown	7 (21.9) 17 (53.1) 8 (25.0)	5 (25.0) 10 (50.0) 5 (25.0)	2 (16.7) 7 (58.3) 3 (25.0)	0.5
**PLT** <207 ≥207 Unknown	11 (34.4) 15 (46.8) 6 (18.8)	8 (40.0) 8 (40.0) 4 (20.0)	3 (25.0) 7 (58.3) 2 (16.7)	0.2

BMI, Body Mass Index; NLR, Neutrophil-to-Lymphocyte Ratio (NLR); PLT, platelet count.

### 3.2 Outcome analysis

**Figure 1:**
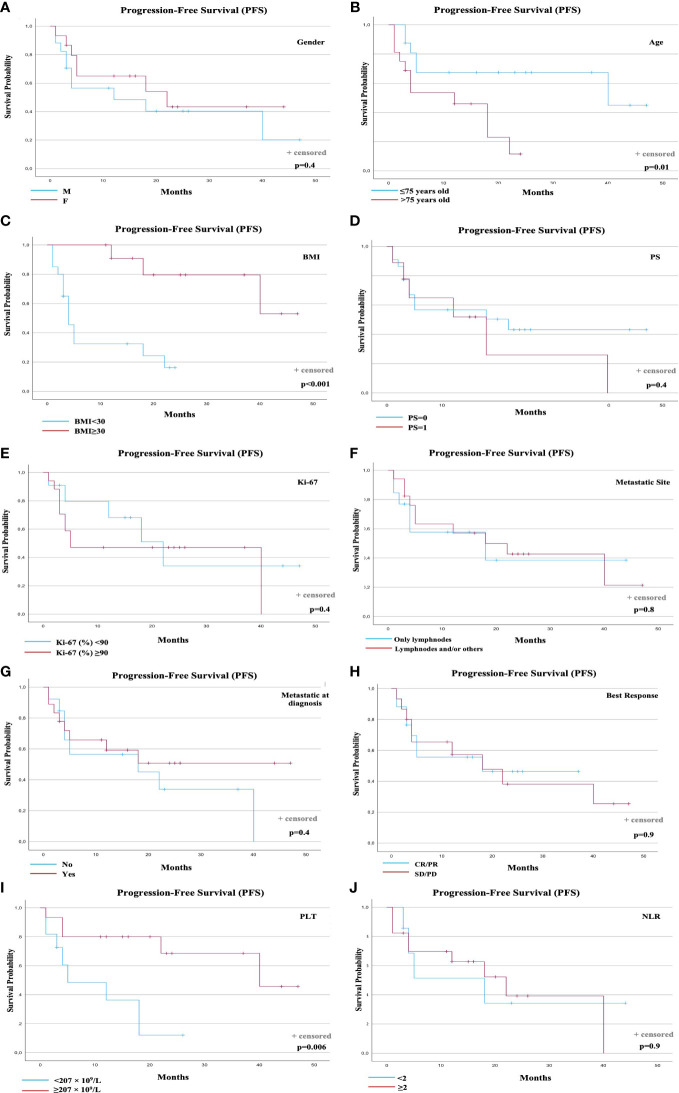
PFS according to prognostic factors. PFS according to: **(A)** Gender; **(B)** Age; **(C)** Baseline BMI; **(D)** Performance status (PS); **(E)** Ki-67 (%); **(F)** Metastatic Sites; **(G)** Metastatic disease at diagnosis; **(H)** Best Response to avelumab; **(I)** Baseline PLT count; **(J)** Baseline NLR. BMI, Body Mass Index; NLR, Neutrophil-to-Lymphocyte Ratio; PLT, Platelets; PFS, Progression-free Survival.

Overall, the median PFS was 18 months (95% confidence interval [CI]: 6.6-31.1). At the time of data analyses, 17 events (progression or death) occurred (53.1%). Notably, the presence of pre-treatment BMI ≥ 30 was significantly associated with longer PFS (**Figure 1C**). A total of 14 events were observed in the group of 20 patients with BMI <30 (70.0%), and 3 events in the group of 12 patients with BMI ≥ 30 (25.0%). Median PFS was 4 months for the BMI < 30 Group (95% CI: 2.5-5.4), and not reached for the group of patients with BMI ≥ 30 (p<0.001). The presence of age <75 years old was significantly associated with longer PFS (p=0.01)(**Figure 1B**). A statistically significant and clinical meaningful correlation between BMI and age was shown using Pearson’s correlation coefficient (p=0.008).

Regarding the outcome data according to PLT count, the pre-treatment value was available for 26 patients. Eight (8) events were observed in the group of 11 patients with PLT count < 207 × 10^9^/L (72.7%), and 5 events in the group of 15 patients with PLT count ≥ 207 × 10^9^/L (33.3%). Median PFS was 10 months for the low PLT Group (95% CI: 4.9, 16.1), and 33 months (95% CI: 24.3, 43.2) for the high PLT Group (p=0.006) (**Figure 1I**). No other prognostic factors (gender, Performance Status, Ki-67 (%), metastatic sites, metastatic stage at diagnosis, best response to avelumab, and NLR at baseline) were statistically associated with PFS (**Figures 1A, D–F, G, H, J**).

**Figure 2:**
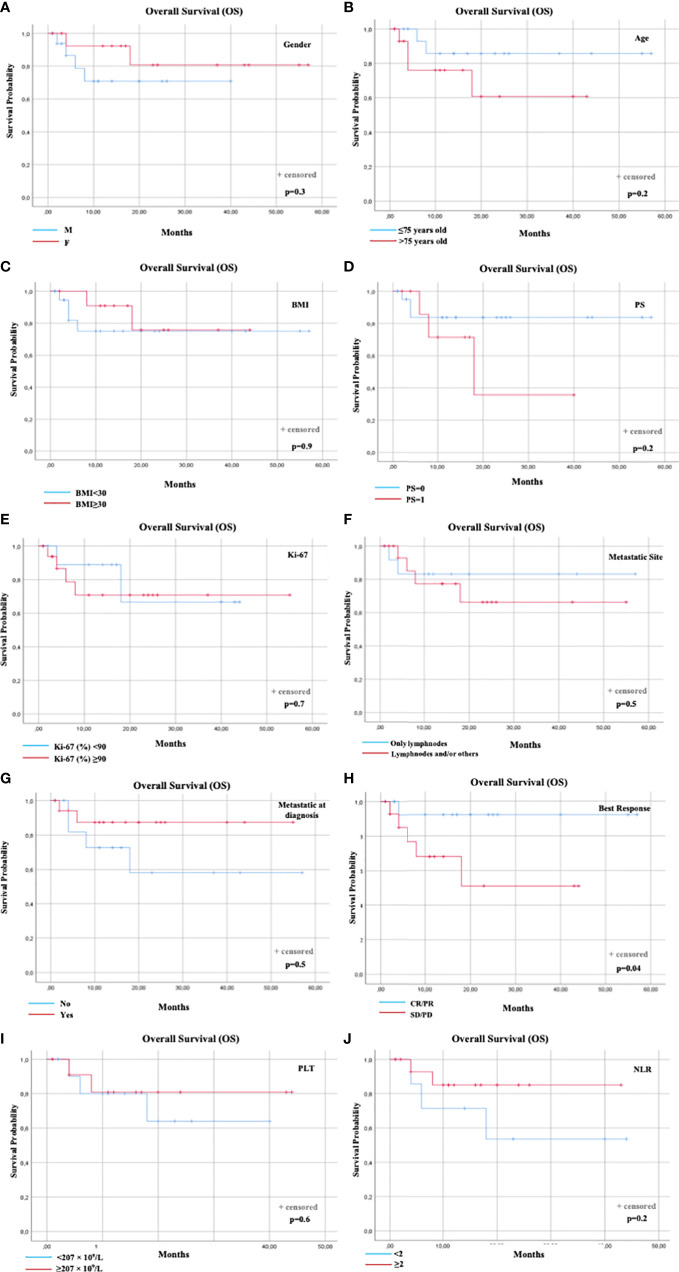
OS according to prognostic factors. OS according to: **(A)** Gender; **(B)** Age; **(C)** Baseline BMI; **(D)** Performance status (PS); **(E)** Ki-67 (%); **(F)** Metastatic Sites; **(G)** Metastatic disease at diagnosis; **(H)** Best Response to avelumab; **(I)** Baseline PLT count; **(J)** Baseline NLR. BMI, Body Mass Index; NLR, Neutrophil-to-Lymphocyte Ratio; PLT, Platelets; Overall Survival.

Overall median OS was not reached. Six (6) total events (deaths) were observed (18.7%). The only prognostic factor statistically associated with OS was the best response to avelumab (p=0.04). The distribution of events according to best response was: 1 event1 in 17 patients with best response CR or PR (5.9%) and 5 events in 15 patients with best response SD or PD (33.3%). Median OS was not reached for the patients with CR/PR or SD/PD (**Figure 2H**). No other prognostic factors were statistically associated with OS (**Figures 1A–G, I, J** **)**.

### 3.3 Objective response rate (ORR) and timing of response

Seventeen (17) out of 32 mMCC patients (53.1%) achieved objective tumor responses, including 3 patients who obtained CRs (9.4%) (**Table 2**). The majority of patients with BMI < 30 achieved a PR (50%) as the best response. In the group with BMI ≥ 30, the SD was the most frequent response achieved (41.7%), followed by PR (33.3%). The median time to objective response was of 4 months (range, 2–7 months) in the overall population. Tumor responses were more rapid in the group of patients BMI < 30 [3.5 months (range, 2-7 months)[ than in those with BMI ≥ 30 [6 months (range, 3-7 months)].

**Table 2:** Objective responses and timing of responses to avelumab.

	All patients	BMI<30	BMI≥ 30
**No. of Patients (%)**	32 (100)	20 (62.5)	12 (37.5)
**Time to** **Response,** **Median (range),** **months**	4 (2-7)	3.5 (2-7)	6 (3-7)
**Overall Response** **(CR + PR),** **No. (%)**	17 (53.1)	12 (60.0)	5 (41.7)
**Best Response, No. (%)** **CR** **PR** **SD** **PD**	3 (9.4) 14 (43.8) 8 (25.0) 7 (21.8)	2 (10.0) 10 (50.0) 3 (15.0) 5 (25.0)	1 (8.3) 4 (33.3) 5 (41.7) 2 (16.7)

CR, complete response; PR, partial response; SD, stable disease; PD, progressive disease.

### 3.4 Univariable and multivariable analysis

**Table 3** summarizes the results of the univariable and multivariable prognostic factor analysis for PFS and OS. Variables included in the univariate analysis were: (1) gender (male or female); (2) age at avelumab start (≤ 75 or > 75 years); (3) pre-treatment BMI (< 30 or ≥ 30); (4) performance status (1 or 0); (5) Ki-67 (%) (<90 or ≥90); (6) metastatic sites (only lymph nodes or lymph nodes and/or other sites); (7) metastatic at diagnosis (no or yes); (8) best response to avelumab (CR/PR or SD/PD); (9) pre-treatment PLT count (< 207 or ≥ 207 × 10^9^/L); (10) pre-treatment NLR (<2 or ≥ 2).

**Table 3:** Univariable and multivariable analysis of prognostic factors for PFS and OS in MCC patients treated with avelumab.

PFS	Univariable Cox Regression		Multivariable Cox Regression	
	HR (95%CI)	*p-value*	HR (95%CI)	*p-value*
Gender (F vs M)	0.67 (0.25-1.78)	NS		
Age (>75 vs ≤75 years old)	3.28 (1.13-9.55)	0.02	1.58 (0.39-6.39)	0.51
BMI (≥30 vs <30)	0.12 (0.03-0.53)	0.005	0.18 (0.03-0.93)	0.04
Performance status (PS) (1 vs 0)	1.47 (0.54-4.02)	NS		
Ki-67 (%) (≥90 vs <90)	1.44 (0.48-4.28)	NS		
Metastatic Sites (Lymphnodes and/or others vs only lymphnodes)	0.90 (0.32-2.51)	NS		
Metastatic at diagnosis (yes vs no)	0.69 (0.26-1.83)	NS		
Best Response (SD/PD vs CR/PR)	1.05 (0.39-2.81)	NS		
PLT (≥207 vs < 207 × 10^9^/L)	0.22 (0.06-0.74)	0.01	0.23 (0.06-0.85)	0.02
NLR (≥2 vs <2)	1.01 (0.31-0.71)	NS		
OS	Univariable Cox Regression		Multivariable Cox Regression	
	HR (95%CI)	*p-value*	HR (95%CI)	*p-value*.
Gender (F vs M)	0.44 (0.08-2.47)	NS		
Age (>75 vs ≤75 years old)	2.81 (0.51-15.39)	NS		
BMI (≥30 vs <30)	0.61 (0.11-3.37)	NS		
Performance status (PS) (1 vs 0)	2.53 (0.50-12.76)	NS		
Ki-67 (%) (≥90 vs <90)	1.36 (0.25-7.46)	NS		
Metastatic Sites (Lymphnodes and/or others vs only lymphnodes)	1.59 (0.29-8.68)	NS		
Metastatic at diagnosis (yes vs no)	0.34 (0.06-1.87)	NS		
Best Response (SD/PD vs CR/PR)	6.41 (0.74-55.34)	0.04		
PLT (≥207 vs < 207 × 10^9^/L)	0.65 (0.11-3.92)	NS		
NLR (≥2 vs <2)	0.35 (0.06-2.12)	NS		

Gender, age, BMI, Performance Status, Ki-67 (%), metastatic sites, metastatic stage at diagnosis, best response to avelumab, PLT and NLR at baseline, were evaluated in the Cox regression model. NS, Not Significant.

Age, BMI, and PLT were found to be statistically significantly associated with PFS in univariable analyses. In the final multivariable Cox regression model, BMI (p=0.04, HR: 0.18), and PLT (p=0.02, HR: 0.23) were significant.

Regarding OS, only best respone to avelumab was statistically significantly associated in univariable analyses. In the final multivariable model, no prognostic factor considered remains statistically significant.

Therefore, these results showed that, in metastatic MCC patients treated with the PD-L1-inhibitor avelumab, BMI ≥ 30 and PLT ≥ 207 × 10^9^/L were significant independent prognostic factors for longer PFS.

PFS and OS curves were plotted according to each prognostic factor (**Figures 1**, **2**).

## 4 Discussion

MCC is an uncommon and highly aggressive neuroendocrine neoplasm difficult to treat (4). Until some years ago the treatment of choice for advanced MCC has been chemotherapy, as well as in poorly differentiated neuroendocrine neoplasms, showing a median PFS of 3–4 months, median OS of less than 10 months, and significant toxicity (4, 18). The association between increased MCC incidence in the elderly and/or immunocompromised subjects and pathological immune dysfunction always suggested a significant role of the immune system in MCC development (19, 20).

Recently, immunotherapy has become the mainstay of treatment of metastatic disease, with durable responses in some cases (21). To date, three immune checkpoint inhibitors (avelumab, pembrolizumab and nivolumab) (7, 22, 23), and the combined nivolumab and ipilimumab (24) resulted to be active in the treatment of advanced MCC. Avelumab was the first approved anti-PD-L1 treatment for patients with metastatic MCC, based on efficacy and safety data observed in the JAVELIN Merkel 200 trial (22). However, as well as for other cancers, only around half of the patients with mMCC respond to the immune‐checkpoint blockade, and a substantial number of patients developed acquired resistance. Consequently, there is a clinical need for predictive factors of immunotherapy response, and understanding the underlying tumor immune escape mechanisms following the ICI treatment is urgently warranted (25).

Recent pan-cancer outcome analyses on patients with or without obesity treated with ICIs showed that presenting with a BMI of 30 or higher was associated with prolonged survival than having BMI less than 30. This study was performed across many cancer types, such as melanoma, NSCLC and SCLC, renal cell carcinoma, bladder cancer, breast cancer, gastric cancer, colorectal cancer, endometrial cancer, sarcoma, and others (12), and the final results were in agreement with some previous published studies that hypothesized the “paradoxical effects” of obesity on cancer immunotherapy across multiple tumor types.

To our knowledge and based on the best data available today, this is the first study that investigates the role of BMI in patients affected by Merkel Cell Carcinoma treated with checkpoint blockade immunotherapy. In our survival analyses, obese mMCC patients showed improved PFS following avelumab treatment than patients with overweight or normal weight. Multivariable Cox regression analyses confirmed these results.

This is consistent with the notion that obesity might lead to an immune aging effect, which could be particularly highlighted in elderly MCC patients. These patients seem to be often immunocompromised, not only for the physiological age-related immune senescence, but also for the frequent history of solid organ and/or hematological stem cell transplantation, and hematological malignancies, especially B cell chronic lymphocytic leukemia (20). Notably, obesity is often accompanied by a chronic low-grade systemic inflammation (26–28), stimulated by the production of the pro-inflammatory cytokines, which alters the microenvironment of expanded adipose tissue. Chronic inflammation can induce immunosuppression as a result of a T cells exhaustion by persistent antigenic stimulation, ultimately inducing dysfunction of the immune system in obese patients (28).

Thus, advanced age, a weakened immune system, and the potential obesity-associated “inflammaging” (28), are key factors that could impact the cancer immune responses in the context of immunotherapy treatment of mMCC patients.

Despite our results showed that most of obese mMCC patients were younger [9 out 12 patients <75 years old (75%) had BMI ≥ 30] than patients with normal weight or overweight, MCC is a tumor of elderly individuals, and the median age of these patients, stratified as younger, were 63 years. In agreement with the known causes of progressive disease or death during avelumab treatment, this result linking higher BMI and younger age was not influenced by clinical factors beyond the metastatic cancer diagnosis.

Although our study focuses on obesity and outcomes of ICI, we further observed a predictive value of platelet counts at baseline before immunotherapy. Interestingly, according to a growing body of evidence, a direct cross-talk between platelets and host immunity seemed to exist (29). Pre-clinical studies have suggested the contribution of platelets to systemic and local responses against cancers (30). Platelets lack a nucleus and DNA available for the transcription of new RNA molecules. However, they can sequester molecules, including RNA and protein transcripts, altering their spliced RNA profiles (31, 32). Specific splice events may be induced in response to signals released by tumor cells and the tumor microenvironment (33). Thus, the resulting “tumor-educated platelets (TEPs) represent dynamic biomolecules with a rich and highly variable repertoire of mRNA, transported from the tumor microenvironment forward multiple anatomic sites (30, 34).

Furthermore, tumor-associated thrombocytosis has been linked to poor clinical outcomes in several cancers, including pancreatic cancer, hepatocellular carcinoma, renal cell carcinoma or glioblastoma (15, 35–38), and it recently has been significantly associated with a poorer prognosis in lung cancer and melanoma patients receiving immunotherapy (39–41). Rachidi et al. (42) showed that platelets can suppress T-cell responses against tumors through the production and activation of immunosuppressive factors, including TGFβ, demonstrating how platelet-related TGFβ activation contributes dominantly to the immunosuppressive tumor microenvironment.

In our study population, we observed that mMCC patients with higher platelet counts assessed at baseline had improved PFS following first-line avelumab treatment. The multivariable model confirmed the independent prognostic value of this finding. The biological reason remains speculative. A possible explanation is that the higher immunosuppression associated with elevated basal platelets may lead to a greater effect of immune checkpoint inhibitors in reinvigorating the T-cell immune response against the tumor following avelumab treatment.

Limitations of our study include the small sample size due to the rarity of this cancer and the retrospective data analysis. On the other hand, all the patients studied were consecutively collected from our single referral Center, thus patients’ data, although retrospective, should be homogenous, limiting possible biases. In addition, BMI is not a perfect assessment of obesity because it cannot distinguish muscle from body fat (43). Accordingly, more specific body fat assessment methods should be used in future prospective studies on larger cohorts.

## 5 Conclusion

MCC has long been considered to be immunogenic cancer because it occurs more frequently and has a worse prognosis in immunosuppressed individuals. Despite the therapeutic impact of reinvigorating the antitumor immune response by immunotherapy, the clinical benefit of ICIs in mMCC is still limited to selected patients. From previous studies across many cancer types, we have learned that in metastatic patients treated with ICIs, obesity is often associated with better clinical outcomes. Our findings validate this concept also in mMCC patients following avelumab treatment, demonstrating a positive effect on PFS of a BMI > 30. The association between basal platelet count and PFS seemed to suggest that a cross-talk platelets-host immunity exists. It remains to be elucidated whether other clinical, immunological or biological factors not yet investigated, may have a further relevant impact on tumor response and clinical outcomes.

## Data availability statement

The original contributions presented in the study are included in the article/supplementary material. Further inquiries can be directed to the corresponding author.

## Ethics statement

The studies involving human participants were reviewed and approved by Ethical committee of the University-affiliated Hospital A.O.U.P. “Paolo Giaccone” (Palermo 1)(approval number 1122). The patients/participants provided their written informed consent to participate in this study.

## Author contributions

The authors confirm contribution to the paper as follows: Study conception and design: LI, VB, AR, and GB. Data collection: AD, LA, LM, IL, RS, AB, TB, FL, MP, DG, IF, FT, AC, and AF. Analysis and interpretation of results: LI, AD, CB, EP, AP, VG, AGi, AGa and GB. Draft manuscript preparation: LI, AD, LM, AP, CB, EP, and GB. All authors contributed to the article and approved the submitted version.

## References

[1] Walsh NM Cerroni L. Merkel cell carcinoma: A review. J Cutan Pathol (2021) 48(3):411–21. doi: 10.1111/cup.13910 33128463

[2] Harms KL Healy MA Nghiem P Sober AJ Johnson TM Bichakjian CK. Analysis of prognostic factors from 9387 merkel cell carcinoma cases forms the basis for the new 8th edition AJCC staging system. Ann Surg Oncol (2016) 23(11):3564–71. doi: 10.1245/s10434-016-5266-4 PMC8881989 27198511

[3] Paulson KG Park SY Vandeven NA Lachance K Thomas H Chapuis AG. Merkel cell carcinoma: Current US incidence and projected increases based on changing demographics. J Am Acad Dermatol (2018) 78(3):457–63.e2. doi: 10.1016/j.jaad.2017.10.028 29102486 PMC5815902

[4] Becker JC Stang A DeCaprio JA Cerroni L Lebbé C Veness M. Merkel cell carcinoma. Nat Rev Dis Prim (2017) 3:17077. doi: 10.1038/nrdp.2017.77 29072302 PMC6054450

[5] Feng H Shuda M Chang Y Moore PS. Clonal integration of a polyomavirus in human merkel cell carcinoma. Science (2008) 319(5866):1096–100. doi: 10.1126/science.1152586 PMC2740911 18202256

[6] Mazziotta C Cervellera CF Lanzillotti C Touzé A Gaboriaud P Tognon M. MicroRNA dysregulations in merkel cell carcinoma: molecular mechanisms and clinical application. J Med Virol (2022) 95(1):e28375. doi: 10.1002/jmv.28375 36477874

[7] Nghiem PT Bhatia S Lipson EJ Kudchadkar RR Miller NJ Annamalai L. PD-1 blockade with pembrolizumab in advanced merkel-cell carcinoma. N Engl J Med (2016) 374(26):2542–52. doi: 10.1056/NEJMoa1603702 PMC4927341 27093365

[8] D'Angelo SP Russell J Lebbé C Chmielowski B Gambichler T Grob JJ. Efficacy and safety of first-line avelumab treatment in patients with stage IV metastatic merkel cell carcinoma: A preplanned interim analysis of a clinical trial. JAMA Oncol (2018) 4(9):e180077. doi: 10.1001/jamaoncol.2018.0077 29566106 PMC5885245

[9] Knepper TC Montesion M Russell JS Sokol ES Frampton GM Miller VA. The genomic landscape of merkel cell carcinoma and clinicogenomic biomarkers of response to immune checkpoint inhibitor therapy. Clin Cancer Res (2019) 25(19):5961–71. doi: 10.1158/1078-0432.CCR-18-4159 PMC6774882 31399473

[10] Giraldo NA Nguyen P Engle EL Kaunitz GJ Cottrell TR Berry S. Multidimensional, quantitative assessment of PD-1/PD-L1 expression in patients with merkel cell carcinoma and association with response to pembrolizumab. J Immunother Canc (2018) 6(1):99. doi: 10.1186/s40425-018-0404-0 PMC6167897 30285852

[11] Zhao X Bao Y Meng B Xu Z Li S Wang X. From rough to precise: PD-L1 evaluation for predicting the efficacy of PD-1/PD-L1 blockades. Front Immunol (2022) 13:920021. doi: 10.3389/fimmu.2022.920021 35990664 PMC9382880

[12] Yoo SK Chowell D Valero C Morris LGT Chan TA. Outcomes among patients with or without obesity and with cancer following treatment with immune checkpoint blockade. JAMA Netw Open (2022) 5(2):e220448. doi: 10.1001/jamanetworkopen.2022.0448 35226089 PMC8886536

[13] Cortellini A Bersanelli M Buti S Cannita K Santini D Perrone F. A multicenter study of body mass index in cancer patients treated with anti-PD-1/PD-L1 immune checkpoint inhibitors: When overweight becomes favorable. J Immunother Canc (2019) 7(1):57. doi: 10.1186/s40425-019-0527-y PMC6391761 30813970

[14] Kichenadasse G Miners JO Mangoni AA Rowland A Hopkins AM Sorich MJ. Association between body mass index and overall survival with immune checkpoint inhibitor therapy for advanced non-small cell lung cancer. JAMA Oncol (2020) 6(4):512–8. doi: 10.1001/jamaoncol.2019.5241 PMC6990855 31876896

[15] Ichihara E Harada D Inoue K Sato K Hosokawa S Kishino D. The impact of body mass index on the efficacy of anti-PD-1/PD-L1 antibodies in patients with non-small cell lung cancer. Lung Canc (2020) 139:140–5. doi: 10.1016/j.lungcan.2019.11.011 31786476

[16] McQuade JL Daniel CR Hess KR Mak C Wang DY Rai RR. Association of body-mass index and outcomes in patients with metastatic melanoma treated with targeted therapy, immunotherapy, or chemotherapy: A retrospective, multicohort analysis. Lancet Oncol (2018) 19(3):310–22. doi: 10.1016/S1470-2045(18)30078-0 PMC5840029 29449192

[17] Spyrou N Vallianou N Kadillari J Dalamaga M. The interplay of obesity, gut microbiome and diet in the immune check point inhibitors therapy era. Semin Cancer Biol (2021) 73:356–76. doi: 10.1016/j.semcancer.2021.05.008 33989733

[18] Nghiem P Kaufman HL Bharmal M Mahnke L Phatak H Becker JC. Systematic literature review of efficacy, safety and tolerability outcomes of chemotherapy regimens in patients with metastatic merkel cell carcinoma. Future Oncol (2017) 13(14):1263–79. doi: 10.2217/fon-2017-0072 PMC6040046 28350180

[19] Kaae J Hansen AV Biggar RJ Boyd HA Moore PS Wohlfahrt J. Merkel cell carcinoma: Incidence, mortality, and risk of other cancers. J Natl Cancer Inst (2010) 102(11):793–801. doi: 10.1093/jnci/djq120 20424236

[20] Harms PW Harms KL Moore PS DeCaprio JA Nghiem P Wong MKK. The biology and treatment of merkel cell carcinoma: Current understanding and research priorities. Nat Rev Clin Oncol (2018) 15(12):763–76. doi: 10.1038/s41571-018-0103-2 PMC6319370 30287935

[21] Gauci ML Aristei C Becker JC Blom A Bataille V Dreno B. Diagnosis and treatment of merkel cell carcinoma: European consensus-based interdisciplinary guideline - update 2022. Eur J Canc (2022) 171:203–31. doi: 10.1016/j.ejca.2022.03.043 35732101

[22] Kaufman HL Russell J Hamid O Bhatia S Terheyden P D'Angelo SP. Avelumab in patients with chemotherapy-refractory metastatic merkel cell carcinoma: A multicentre, single-group, open-label, phase 2 trial. Lancet Oncol (2016) 17(10):1374–85. doi: 10.1016/S1470-2045(16)30364-3 PMC5587154 27592805

[23] Walocko FM Scheier BY Harms PW Fecher LA Lao CD. Metastatic merkel cell carcinoma response to nivolumab. J Immunother Canc (2016) 4:79. doi: 10.1186/s40425-016-0186-1 PMC5109712 27879975

[24] Kim S Wuthrick E Blakaj D Eroglu Z Verschraegen C Thapa R. Combined nivolumab and ipilimumab with or without stereotactic body radiation therapy for advanced merkel cell carcinoma: A randomised, open label, phase 2 trial. Lancet (2022) 400(10357):1008–19. doi: 10.1016/S0140-6736(22)01659-2 PMC9533323 36108657

[25] Russo A Incorvaia L Malapelle U Del Re M Capoluongo E Vincenzi B. The tumor-agnostic treatment for patients with solid tumors: A position paper on behalf of the AIOM- SIAPEC/IAP-SIBioC-SIF Italian scientific societies. Crit Rev Oncol Hematol (2021) 165:103436. doi: 10.1016/j.critrevonc.2021.103436 34371157

[26] Santoni M Massari F Bracarda S Procopio G Milella M De Giorgi U. Body mass index in patients treated with cabozantinib for advanced renal cell carcinoma. A New Prognost Factor? Diagnost (Basel) (2021) 11(1):138. doi: 10.3390/diagnostics11010138 PMC7831923 33477676

[27] Vincenzi B Badalamenti G Armento G Silletta M Spalato Ceruso M Catania G. Body mass index as a risk factor for toxicities in patients with advanced soft-tissue sarcoma treated with trabectedin. Oncology (2018) 95(1):1–7. doi: 10.1159/000487266 29510410

[28] Wang Z Aguilar EG Luna JI Dunai C Khuat LT Le CT. Paradoxical effects of obesity on T cell function during tumor progression and PD-1 checkpoint blockade. Nat Med (2019) 25(1):141–51. doi: 10.1038/s41591-018-0221-5 PMC6324991 30420753

[29] Honrubia-Peris B Garde-Noguera J García-Sánchez J Piera-Molons N Llombart-Cussac A Fernández-Murga ML. Soluble biomarkers with prognostic and predictive value in advanced non-small cell lung cancer treated with immunotherapy. Cancers (Basel) (2021) 13(17):4280. doi: 10.3390/cancers13174280 34503087 PMC8428366

[30] Dong X Ding S Yu M Niu L Xue L Zhao Y. Small nuclear RNAs (U1, U2, U5) in tumor-educated platelets are downregulated and act as promising biomarkers in lung cancer. Front Oncol (2020) 10:1627. doi: 10.3389/fonc.2020.01627 32903345 PMC7434840

[31] Nilsson RJ Balaj L Hulleman E van Rijn S Pegtel DM Walraven M. Blood platelets contain tumor-derived RNA biomarkers. Blood (2011) 118(13):3680–3. doi: 10.1182/blood-2011-03-344408 PMC7224637 21832279

[32] Sol N Wurdinger T. Platelet RNA signatures for the detection of cancer. Cancer Metastasis Rev (2017) 36(2):263–72. doi: 10.1007/s10555-017-9674-0 PMC5557864 28681241

[33] Leto G Incorvaia L Flandina C Ancona C Fulfaro F Crescimanno M. Clinical impact of cystatin C/Cathepsin l and Follistatin/Activin a systems in breast cancer progression: A preliminary report. Cancer Invest (2016) 34(9):415–23. doi: 10.1080/07357907.2016.1222416 27636861

[34] Best MG Wesseling P Wurdinger T. Tumor-educated platelets as a noninvasive biomarker source for cancer detection and progression monitoring. Cancer Res (2018) 78(13):3407–12. doi: 10.1158/0008-5472.CAN-18-0887 29921699

[35] Lu L Su Z Zheng P Wu Z Zhang Y He H. Association between platelet count and hepatocellular carcinoma overall survival: A large retrospective cohort study. BMJ Open (2020) 10(11):e038172. doi: 10.1136/bmjopen-2020-038172 PMC7651714 33158820

[36] Brown KM Domin C Aranha GV Yong S Shoup M. Increased preoperative platelet count is associated with decreased survival after resection for adenocarcinoma of the pancreas. Am J Surg (2005) 189(3):278–82. doi: 10.1016/j.amjsurg.2004.11.014 15792750

[37] Brockmann MA Giese A Mueller K Kaba FJ Lohr F Weiss C. Preoperative thrombocytosis predicts poor survival in patients with glioblastoma. Neuro Oncol (2007) 9(3):335–42. doi: 10.1215/15228517-2007-013 PMC1907417 17504931

[38] Inoue K Kohashikawa K Suzuki S Shimada M Yoshida H. Prognostic significance of thrombocytosis in renal cell carcinoma patients. Int J Urol (2004) 11(6):364–7. doi: 10.1111/j.1442-2042.2004.00808.x 15157203

[39] Wang H Li C Yang R Jin J Liu D Li W. Prognostic value of the platelet-to-lymphocyte ratio in lung cancer patients receiving immunotherapy: A systematic review and meta-analysis. PloS One (2022) 17(5):e0268288. doi: 10.1371/journal.pone.0268288 35522679 PMC9075650

[40] Lu X Wan J Shi H. Platelet-to-lymphocyte and neutrophil-to-lymphocyte ratios are associated with the efficacy of immunotherapy in stage III/IV non-small cell lung cancer. Oncol Lett (2022) 24(2):266. doi: 10.3892/ol.2022.13386 35782904 PMC9247654

[41] Guida M Bartolomeo N Quaresmini D Quaglino P Madonna G Pigozzo J. Basal and one-month differed neutrophil, lymphocyte and platelet values and their ratios strongly predict the efficacy of checkpoint inhibitors immunotherapy in patients with advanced BRAF wild-type melanoma. J Transl Med (2022) 20(1):159. doi: 10.1186/s12967-022-03359-x 35382857 PMC8981693

[42] Rachidi S Metelli A Riesenberg B Wu BX Nelson MH Wallace C. Platelets subvert T cell immunity against cancer *via* GARP-TGFβ axis. Sci Immunol (2017) 2(11):eaai7911. doi: 10.1126/sciimmunol.aai7911 28763790 PMC5539882

[43] Incorvaia L Rinaldi G Badalamenti G Cucinella A Brando C Madonia G. Prognostic role of soluble PD-1 and BTN2A1 in overweight melanoma patients treated with nivolumab or pembrolizumab: Finding the missing links in the symbiotic immune-metabolic interplay. Ther Adv Med Oncol (2023) 15(15):17588359231151845. doi: 10.1177/17588359231151845 PMC9936535 36818688

